# Evaluation of three Polymerase chain reaction tests targeting morphological transforming region II, UL-83 gene and glycoprotein O gene for the detection of Human Cytomegalovirus genome in clinical specimens of immunocompromised patients in Chennai, India

**DOI:** 10.1186/1743-422X-3-20

**Published:** 2006-03-30

**Authors:** P Sowmya, HN Madhavan, KL Therese

**Affiliations:** 1 L & T Microbiology Research Center, Vision Research Foundation, 18, College Road, Chennai - 600-006, India

## Abstract

**Background:**

Human Cytomegalovirus (HCMV) continues to be an important cause of morbidity and occasional mortality in immunocompromised patients. Polymerase chain reaction (PCR) is the most sensitive and commonly used method for the assessment of HCMV infection in the immunocompromised patients at risk from severe associated clinical manifestations. However, there is little consistency in the qualitative PCR used for different regions of HCMV genome. Therefore, the performance of three Qualitative PCR tests to detect HCMV genome in clinical specimens from immunocompromised patients was evaluated. With pp65 antigenemia assay as the "gold standard", nested PCR for morphological transforming region II (mtr II) and glycoprotein O (gO) gene and uniplex PCR for UL 83 gene were applied on 92 consecutive clinical specimens obtained from 74 immunocompromised patients with clinically suspected HCMV disease. Virus isolation was attempted on 12 clinical specimens from six pp65 antigenemia positive patients. Based on the pp 65 antigenemia results as "gold standard", the sensitivity, specificity, positive predictive value and negative predictive value for each PCR was calculated.

**Results:**

The PCR targeting mtr II region showed a higher sensitivity (100%) and negative predictive value (100%) than the other two PCRs in detecting HCMV DNA from clinical specimens obtained from different immunocompromised patient population of Chennai region, India.

**Conclusion:**

The results suggests that the optimal method of detection of HCMV DNA could be achieved by PCR using primer sequences targeting mtr II region of genome of HCMV in Chennai region, India.

## Background

Human Cytomegalovirus (HCMV), a widespread Herpes virus, usually produces asymptomatic infections in immunocompetent hosts. Serious disease can occur in immunocompromised individuals and in congenitally infected newborns. Symptoms in these patients range from a mild disease to life threatening multiorgan system disease. Conventional methods for laboratory diagnosis of CMV infection include serology, virus culture by conventional tube method or rapid shell vial assay and antigen detection. Culture is the "gold standard" but is a relatively insensitive laboratory method and serology results are difficult to interpret especially in immunocompromised patients [1-3]. pp65 antigenemia assay is used as a test for monitoring those at higher risk of developing CMV disease and to initiate pre-emptive therapy [4,5].

Detection of HCMV DNA in clinical specimens by nucleic acid based amplification methods such as Polymerase chain reaction (PCR) contributes to a rapid and early diagnosis [6-8]. Primer pairs for the detection of the genes coding for the Immediate Early (IE) antigen and Late antigen (LA) were initially used for the detection of HCMV genome in urine and peripheral blood leucocyte specimens [9,10]. Since then, a variety of primer pairs are being used for routine diagnosis of HCMV infection in various patient populations. Sequence variations in the viral genome have been shown to affect the ability of the PCR using different primer sets to detect HCMV DNA [11-14]. Little is known about the sequence variations in the regions of HCMV genome under the present study viz: morphological transforming region II (mtr II), UL 83 and glycoprotein O (gO) gene. The PCRs for the aforementioned regions were already standardized in our laboratory using cultures of CMV AD-169 strain (ATCC VR-538). pp65 antigenemia assay is a rapid, reliable and superior to both rapid shell vial assay and conventional test tube culture in the detection of HCMV in the clinical specimens from immunocompromised patients indicating active HCMV disease. [15,16]. Therefore, the antigenemia assay was considered as "gold standard" in the present study to evaluate the efficacy of the three Polymerase chain reaction tests to detect HCMV genome in the clinical specimens of clinically suspected HCMV disease in immunocompromised patients in Chennai, India.

## Results

Of the total 92 specimens from 74 patients tested, the pp 65 antigenemia was present in 48 clinical specimens from 38 patients and these were considered as positive for the "gold standard", definition for active CMV disease. The patients in whom pp65 antigenemia was positive presented a mean of 44.7 positive cells in the antigenemia assay (range 13 – 162 cells). HCMV was isolated from three clinical specimens (one peripheral blood leucocyte and two urine) from three patients, positive for pp65 antigenemia. Of the total 48 clinical specimens positive by the "gold standard", when tested by PCR methods all were positive for mtr II region, 27 for UL -83 gene and 21 for gO gene. This increase in clinical sensitivity by the PCR for mtr II over the UL 83 and gO PCRs were 44% and 56% respectively. The increase in the clinical sensitivity of the PCR for mtr II was statistically significant (P < 0.0001 by Fisher's exact test for two proportions). However, the difference in the clinical specificity between the three PCR tests were not statistically significant (P >0.08, by Fisher's exact test for two proportions). A summary of the results for the clinical specimens is presented in Table 2. All the 45 peripheral blood leucocytes obtained from the controls (seropositive healthy donors) remained negative by pp65 antigenemia assay and all the three PCR tests for HCMV.

## Discussion

Human Cytomegalovirus has long been recognized as a major cause of life- threatening complications in immunosuppressed individuals. There is perceived need for the use of a reliable technique that allows an early detection of the viral activation to help decide on early use of pre-emptive therapy in those at greater risk of the disease. Technique such as virus isolation though most specific cannot be practiced on a regular basis due to lack of its sensitivity and non-availability of human diploid fibroblasts in this part of the world. Quantitative pp 65 antigenemia, used to monitor and detect CMV disease, is well established to have a higher positive predictive value for the disease [3]. Since antigenemia is cell based and a low frequency event, a sufficient number of granulocytes are necessary for a reliable result [3]. This becomes difficult in Bone marrow transplant or other patients with severe leucopenia. Other difficulties include the necessity for immediate processing of the specimen (within 6 hrs, stored specimens may give erroneous results), difficulty in processing a large number of specimens at a time and subjective component in slide reading, which requires expertise [3]. The drawbacks of pp65 antigenemia assay or virus isolation may be overcome by the use of rapid, sensitive and normalized method such as PCR for the detection of HCMV genome in the clinical specimens, which can be applied on a large scale of clinical specimens without any difficulty in a standard laboratory for routine diagnosis of HCMV infection.

Amplification of HCMV genome by PCR is a rapid and sensitive method for detection of HCMV in clinical specimens. The choice of PCR primers for HCMV genome detection in a clinical specimen is crucial since the genome of HCMV is reported to be highly variable [11-14]. The primers targeting regions such as the Major immediate early gene exon 4, regions of gene coding late antigen, glycoprotein B (gB) and glycoprotein H (gH) which are widely used have failed to detect the HCMV genome in certain clinical specimens due to primer target mismatch owing to the large sequence variations in HCMV genome. Nucleotide substitutions and even deletions of certain ORFs have been found along the genome of HCMV [17]. Studies by Distefano *et al*., suggested that PCR for gB gene was more reliable than the Major immediate early gene exon 4 or Late antigen gene in detecting HCMV genome in the clinical specimens from congenital and perinatal infections in Argentina [13]. Studies by Wirgart *et al*. suggested that the DNA polymerase gene and gB gene were more conserved and can be used for diagnosis of HCMV infections in different patient populations [14]. PCRs for gB and gH genes of HCMV were compared in a Brazilian study on the renal transplant recipients by Aquino and Figueiredo where in a multiplex format of both the genes was suggested for reliable detection of HCMV genome [18]. PCRs for MIE gene (IE1), glycoprotein B (gp 58) and structural phosphoprotein (pp 150) were compared with pp 65 antigenemia in heart and lung transplant recipients by Barber *et al*. where in all the PCR showed a high sensitivity of 100% though gp 58 was associated more with a positive PCR signal than the other two PCRs [19].

There are only a few reports available on the primers targeting the regions of HCMV genome under the present study [20-22]. Therefore, the efficacy of these three primers were evaluated on different clinical specimens obtained from different high risk immunocompromised patient populations against pp65 antigenemia assay as gold standard. Blood from the healthy seropositive controls did not yield any detectable CMV DNA by any of the three PCRs following amplification. Thus, though the PCRs were considered as highly sensitive methods, they did not detect low – level latent HCMV infection present in the healthy immunocompetent individuals in this study.

pp65 antigenemia assay detected active CMV disease in 38 of 74 patients. The virus isolation had a low sensitivity, positive only in three out of 12 patients with CMV disease as evidenced by a positive antigenemia and these were positive by PCR for all the three regions. We hypothesise that PCRs for all the three regions may become positive only with a high viral load.

The failure of the PCR targeting UL 83 may be due to its lower analytical sensitivity, as it is a uniplex PCR. The PCR for gO gene though a nested PCR, shows a lower clinical sensitivity than the uniplex PCR for UL 83 and this may be attributed to the strain variations in gO gene leading to the primer target mismatch and hence loss of an amplification signal.

## Conclusion

Results of our study showed that the PCR for mtr II had 100% sensitivity, 100 % negative predictive value, 87% positive predictive value and 84% specificity. Therefore, it is the most suitable for routine use in Chennai region in India.

## Methods

### Study design

Clinical specimens were investigated at L & T Microbiology Research Centre, Vision Research Foundation, in Sankara Nethralaya, Chennai, India during December 2004 to July 2005 for the possible association of CMV infection in 74 immunocompromised patients. Clinical specimens were investigated because of clinical suspicion of CMV- related disease in these patients. The patients with pp65 antigenemia positivity were defined to have activation of CMV disease and this test was used as the "gold standard" to evaluate the three PCRs for their clinical specificity, sensitivity and predictive values [20-22].

### Patients and specimens

The distribution of the clinical specimens in relation to the clinical status of the patients from whom they were collected is provided in Table 1. In total, 92 specimens [74 blood, 18 urine] from 74 patients were analyzed during the course of the study. In addition blood samples from 45 healthy blood donors with no history or recent CMV infection but seropositive for CMV were used as controls for all the tests. Among the 74 patients, 47 were males and 27 females. The age of the patients ranged from 40 hours after birth to 67 years. The patients clinically presented with multiple symptoms such as fever, jaundice and leucopenia. Three of the renal transplant recipients had a clinical evidences of a moderate graft rejection. CMV retinitis was predominant in the HIV infected individuals.

**Table 1:** Distribution of 92 clinical specimens in relation to the clinical status of the 74 patients with suspected CMV infections.

**Clinical Status of the Patients**	**Total number of patients (n = 74)**	**Clinical Specimens collected (n= 92)**	
		
		**Blood only (n= 56)**	**Blood and urine (n = 18 × 2 : 36)**
Solid organ transplantation	59 (79.7%)	43	16
Bone marrow transplantation	3 (4.0%)	3	-
HIV infected individuals	7(9.5%)	7	-
Congenital/neonates	5(6.8%)	3	2

**Table 2:** Results of the three PCRs for the diagnosis of suspected CMV infections in comparison to pp65 antigenemia (gold standard)^a^.

**Diagnostic test result**	**pp65 Positives (n = 48)**				**pp65 Negatives (n = 44)**			
	
	**No. of Clinical specimens**	**SENS (%)**	**PPV (%)**	**FN (%)**	**No. of Clinical specimens**	**SPEC (%)**	**NPV (%)**	**FP (%)**
PCR for mtrII positive	48	100	87	0	7	84	100	14
PCR for mtr II negative	0				37			
PCR for UL 83 positive	27	56	87	44	4	91	66	8
PCR for UL 83 negative	21				40			
PCR for gO positive	21	44	91	56	2	95	61	4
PCR for gO negative	27				42			

^a^Abbreviations: SENS, sensitivity; PPV, Positive predictive value; FN, False negative; SPEC, specificity; NPV, Negative predictive value; FP, False positive

### Virus isolation

Virus isolation was performed on 12 clinical specimens (peripheral blood leucocytes and urine specimens of six patients) using rapid shell vial technique. Human Tenon's capsule fibroblasts grown on cover slips were inoculated with the clinical specimens. For inoculation purposes, 100 μl of peripheral blood leukocyte suspension of blood specimens and 100 μl of decontaminated centrifuged deposits of the urine specimens were used. The cover slips were stained at 48 h with mouse monoclonal antibody (DAKO, A/S, Denmark) raised against the early antigen of HCMV and rabbit anti-mouse fluorescein thiocyanate conjugate (DAKO, A/S, Denmark). One or more positive fluorescent nuclei indicated positive result [20].

### pp65 antigenemia assay

pp65 antigenemia for CMV was carried out on 5 ml of EDTA anticoagulated blood within six hours of receipt of the specimen as described previously with few modifications [23]. In brief, Cytospin smear made with the 2 × 10^5 ^leucocytes obtained from EDTA anticoagulated blood after dextran sedimentation and erythrolysis using 0.8% ammonium chloride was fixed in methanol for 10 minutes. The cells were permeabilized using 0.5% Non-idet P40. The smears were stained with mouse monoclonal antibody (DAKO, A/S, Denmark) raised against the pp65 antigen of HCMV and rabbit anti-mouse fluorescein thiocyanate conjugate (DAKO, A/S, Denmark). 0.5 % Evan's blue (Himedia, India), was used as a counter stain. The smears were examined under fluorescent microscope (Optiphot, Nikon, Japan). A positive assay result was defined by the presence of at least 1 positively stained leukocyte on the slide, and the result was expressed as the number of CMV pp65-positive cells per 2 × 10^5 ^leukocytes.

### PCR amplification of the three target regions

The extraction of the DNA was performed using two commercially available DNA extraction columns strictly adhering to the manufacturer's instruction. For blood specimens, 100 μl of the buffy coat was extracted using QIAamp DNA mini Kit (QIAGEN, Germany). For urine and culture harvests Biogene DNA extraction kit, (BIOGENE Reagents Inc, CA, USA) was used. The PCR for detection of mtrII region was performed as described [20]. The nested primers of mtr II, CMTR 1-5'-CTG TCG GTG ATG GTC TCT TC-3' and CMTR 2-5'-CCC GAC ACG CGG AAA AGA AA-3' for the first round and CMTR3 5' TCT CTG GTC CTG ATC GTC TT-3' and CMTR4-5'-GTG ACC TAC CAA CGT AGG TT-3' for the second round generated 234 bp and 168 bp products respectively The PCR for detection of gO gene was performed as previously described [21]. The nested primers of gO, gO 1-5'-CAG CTT CGA AAA CCG GCC AAA TAC G-3' and gO 2-5'-AAT ATA CTT GGG GAC GCG AAA TAG A-3' for the first round generated a 375 bp and gO3-5'-GCT TCG AAA ACC GGC CAA ATA CG-3' and gO4-5'-ATA CTT GGG GAC GCG AAA TAG A-3' for the second round generated a 370 bp product. The primers of the UL 83 PCR, which were originally designed for application in 5' nuclease, assay and were adapted for standard uniplex PCR by us [22]. The uniplex primers of UL-83, UL83 1-5'-GGG ACA CAA CAC CGT AAA GC-3' and UL83 2-5'-GTC AGC GTT CGT GTT TCC CA-3' generated a 283 bp product. Analysis of the PCR products (10 μl) was undertaken by electrophoretic separation on an ethidium bromide-stained 2% agarose gel. The analytical sensitivity of the PCRs for mtr II, UL 83 and gO gene were determined by application of the individual PCRs on log dilutions of AD169 DNA and was found to be 1 fg, 50 fg and 500 fg of HCMV DNA respectively.

### Statistical analysis

Diagnostic data from clinical specimens of 74 patients with suspected CMV infections were used for the determination of the clinical sensitivity, specificity, positive predictive values and negative predictive values of the three different PCR results using pp65 antigenemia and/or culture results as the "gold standard"[24]. The difference in the clinical sensitivity and the specificity of the three different PCRs were statistically analyzed by Fisher's exact test for two proportions.

## Competing interests

The author(s) declare that they have no competing interests.

## Authors' contributions

The corresponding author, H.N. Madhavan is the principal investigator of the study; is involved in the design, supervision, data analyses and writing of the report. P. Sowmya performed all the virological investigations, nucleotide sequencing and analyses of data. All authors were involved in the preparation of this "*Research Article*".
